# Hydroxychloroquine in IgA nephropathy: a systematic review

**DOI:** 10.1080/0886022X.2021.2000875

**Published:** 2021-11-15

**Authors:** Gabriel Stefan, Gabriel Mircescu

**Affiliations:** a Department of Nephrology, “Carol Davila” University of Medicine and Pharmacy, Bucharest, Romania; b “Dr. Carol Davila” Teaching Hospital of Nephrology, Bucharest, Romania

**Keywords:** eGFR, hydroxychloroquine, IgA nephropathy, meta-analysis, proteinuria, systematic review

## Abstract

**Background:**

Hydroxychloroquine (HCQ) has recently been reported to be a promising and safe anti-proteinuric agent for IgA nephropathy (IgAN) patients. In the present systematic review, we aimed to summarize the evidence concerning the benefits and risks of HCQ therapy in IgAN.

**Methods:**

Electronic databases were searched for randomized, cohort, or case-control studies with IgAN biopsy-proven patients comparing the effects of HCQ with angiotensin-converting enzyme inhibitors/angiotensin receptor blockers or immunosuppression on proteinuria reduction.

**Results:**

Five studies, one randomized and three observational, involving a total of 504 patients, were eligible for inclusion. Overall, there was a tendency of HCQ treatment to reduce proteinuria. In the studies where the control arm was supportive therapy, HCQ significantly reduced proteinuria at 6 months. However, in the studies that compared HCQ to immunosuppressive therapy, we found no difference in proteinuria reduction. HCQ had no impact on eGFR.

**Conclusion:**

HCQ seems to be an efficient alternative therapy for patients with IgAN who insufficiently respond to conventional therapy. However, ethnically diverse randomized controlled studies with long-term follow-up are needed.

## Introduction

Immunoglobulin A (IgA) nephropathy (IgAN) is the most prevalent cause of primary glomerulopathies in the developed world [1,2].

The mainstay of treatment for IgAN patients consists of the renin-angiotensin-aldosterone system inhibitors (RAASIs) in patients with proteinuria higher than 0.5–1 g/day or hypertension [3–6]. The latest randomized controlled trials have shown significantly better kidney survival in patients on such therapy as long as the targets for proteinuria and blood pressure are attained [5,7,8]. However, the utility of corticosteroids or immunosuppression is still a matter of controversy due to the highly variable spectrum of IgAN and the safety issues related to this class of medication [7,8]. Therefore, other treatment options are needed.

Hydroxychloroquine (HCQ), a classical antimalarial drug, with immunomodulatory and anti-inflammatory characteristics, has been widely used for the treatment of autoimmune diseases, like systemic lupus erythematosus and rheumatoid arthritis [9].

Recent studies reported that HCQ therapy in patients with IgAN effectively reduced proteinuria [10,11]. In comparison with conventional RAASIs alone, HCQ markedly reduced proteinuria after 6 months [11]. However, compared with corticosteroid therapy, HCQ was similarly or less effective in lowering proteinuria, but had fewer adverse events [12,13]. Large multicentric trials on different ethnic patients with IgAN are lacking. Therefore, we aimed to perform a systematic review to evaluate the efficacy and safety of HCQ in IgAN.

## Methods

### Study protocol

We have conducted a systematic review in accordance with the Cochrane-based methodology [14]. All the studies that aimed to assess the efficacy and safety of HCQ in patients with IgAN were eligible for inclusion. The data were extracted according to the methodology, and the manuscript was reviewed by the Ethics Committee of the "Dr Carol Davila" Teaching Hospital of Nephrology. However, since no experimental interventions were performed, the present systematic review and meta-analysis was not under any guideline specification, legislations, or permissions from the Ethics Committee.

### Search strategy

Electronic databases – PubMed, EMBASE, ISI Web of Science, Medline/Ovid, Google Scholar, Cumulative Index to Nursing and Allied Health Literature (CINAHL), and Cochrane Database of Systematic Review – were searched using the terms: hydroxychloroquine, chloroquine, plaquenil, and IgA nephropathy, glomerulonephritides IgA, IgA glomerulonephritis, immunoglobulin A nephropathy, Berger’s disease.

We limited our search to studies on human subjects published in peer-reviewed journals from January 1985 to January 2021.

### Study selection

Eligible studies satisfied the following inclusion criteria: (i) population: patients with biopsy-proven IgAN; (ii) study design: randomized, cohort, or case-control studies; (iii) intervention: hydroxychloroquine; (iv) comparison: angiotensin-converting enzyme inhibitors/angiotensin receptor blockers (ACEI/ARB) or immunosuppression; (v) outcome: the primary outcome was change in proteinuria from baseline to end of the study, and the secondary outcome was change in eGFR during follow-up.

Studies were excluded if: (i) they were systematic reviews, comments, case reports, conference abstracts, and editorials; (ii) study subjects with secondary IgAN; (iii) manuscript was not written in English.

### Data extraction, validity assessment, and quality assessment

**Figure 1.**
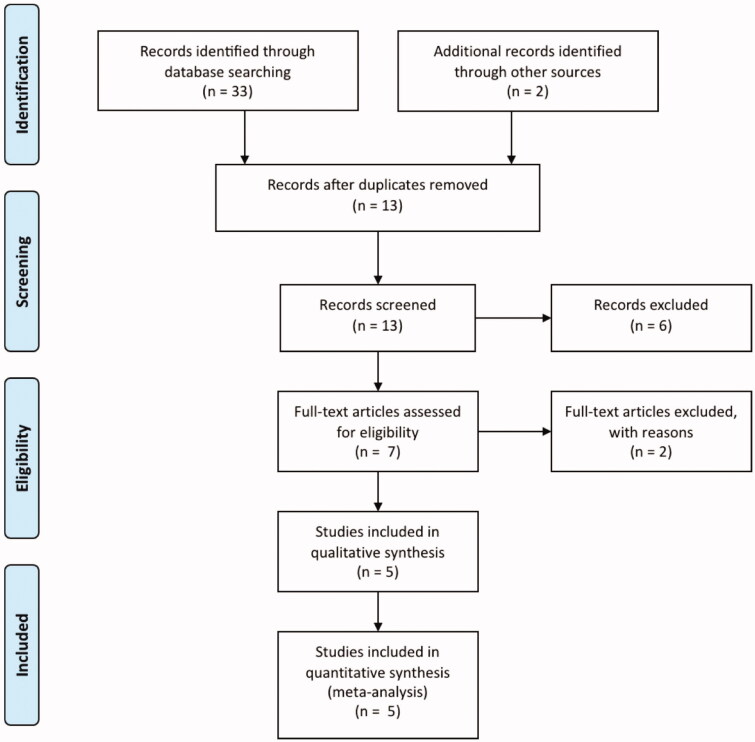
Study selection process of the systematic review (PRISMA diagram, Preferred Reporting Items for Systematic Reviews and Meta-Analyses).

The titles and abstracts were screened independently by two authors. Two authors independently assessed the full text of the included studies to determine which of them satisfy the inclusion criteria. Differences between the two authors were settled by discussion (Figure 1).

The quality assessment of the selected studies for inclusion was done using the Jadad score. The Jadad score consisted of three items: randomization (0–2 points), blinding (0–2 points), and dropouts and withdrawals (0–1 points). The response to each item was either “yes” (1 point) or “no” (0 points). The final score ranged from 0 to 5 points, with higher scores indicating better reporting [15].

### Statistical analysis

The systematic review was performed with The Cochrane Collaboration Review Manager software package (RevMan Version 5.4).

## Results

### Selection and description of studies

We included in our final analysis five studies - one randomized and three observational - involving a total of 504 patients (minimum 28 and maximum 184 patients) (Table 1).

**Table 1.** Characteristics of the studies included in the systematic review.

Reference	Study design, follow-up time, N	Jadad score	RASI	Intervention	Control	Baseline proteinuria (g/d)		End of study proteinuria (g/d)		Baseline eGFR (mL/min)		End of study eGFR (mL/min)	
						HCQ	Control	HCQ	Control	HCQ	Control	HCQ	Control
Gao, 2017 [10]	prospective, paired case-control study. 24 weeks. 14 HCQ/14 control.	1	Both groups	HCQ + Losartan	RASI (Losartan)	0.9 ± 0.4	0.7 ± 0.2	0.5 ± 0.2*	0.7 ± 0.3*	83 ± 18	84 ± 19	81 ± 21	81 ± 19
Liu, 2019 [11]	RCT, double-blind, placebo controlled. 6 months. 30 HCQ/30 control	3	Both groups	HCQ + RASI	RASI	1.6 [1.1, 2.2]	1.9 [1.3, 2.6] g/d	0.9* [0.6, 1.0] g/d	1.9* [0.9, 2.6] g/d	52.1 ± 19.7	55.5 ± 18.7	53.1 ± 20.2	55.2 ± 19.7
Yang, 2019 [13]	retrospective, case-control PPS. 6 months. 92 HCQ/92 control	0	Both groups	HCQ	CS therapy	1.7 [1.2, 2.3]	1.8 [1.3, 2.5]	0.8 * [0.6, 1.1]	0.7 * [0.3, 1.1]	56.8 ± 20.4	55.2 ± 22.9	57.1 ± 20.2	56.1 ± 21.9
Tang, 2020 [12]	retrospective, case-control PPS. 6 months. 26 HCQ/26 control	0	Both groups	HCQ + IS therapy	IS therapy	2.3 [1.4, 2.9]	2.3 [1.5, 2.9]	1.1 [0.8, 1.6]	1.2 [0.8, 2.5]	47.6 [34.6, 67.4]	51.5 [26.4, 82.3]	50.9 [30.9, 71.1]	56.3 [19.3, 79.2]
Tang, 2021 [19]	retrospective, cohort. 2 years. 180 patients	0		HCQ	–	1.6 [1.2, 2.3]	–	1.0 [0.5, 1.7]	–	65.8 ± 25.2	–	63.9 ± 25.9	–

Bd: two times a day; CS: corticosteroids; HCQ: hydroxychloroquine; IS: immunosuppressive; od: one time a day; PPS: propensity matched score; RASI: renin angiotensin system inhibitors; RCT: randomized controlled trial; td: three times a day.

*Statistically significant.

We analyzed as primary endpoint the change in proteinuria from baseline to end of study, and as secondary endpoint we evaluated the change in eGFR.

Hydroxychloroquine was the intervention agent in all the included trials; however, in two studies HCQ was compared to immunosuppressive therapy [10,11], while in the other two it was compared to supportive therapy (Table 1) [12,13].

Liu et al performed a double-blind, randomized, placebo-controlled trial, in which IgAN patients on optimized RAASI therapy were randomly assigned to receive oral HCQ or placebo for 6 months [11]. Similarly, Gao et al. conducted a prospective, paired case-control study in which IgAN patients were assigned to receive HCQ and losartan versus losartan only for 24 weeks [10]. Of note, patients from the first study did not receive any immunosuppression treatment in the previous year and those in the second study during the previous three months (Table 1).

In a retrospective, case-control, propensity-matched study, Yang et al. compared IgAN patients on HCQ plus RAASI versus corticotherapy for 6 months [13]. In a similar study design, Tang et al. compared patients on immunosuppression therapy and HCQ versus conventional immunosuppression (Table 1) [12].

The HCQ dose varied according to the baseline eGFR. Thus, the dose was 200 mg twice daily for patients with an eGFR greater than 60 mL/min, 100 mg two or three times daily for patients with an eGFR between 30 and 59 mL/min and 100 mg once daily for patients with an eGFR between 15 and 29 mL/min.

### Quality assessment of the included studies

We included in the analysis one randomized and four observational studies. Therefore, only one study was assessed as being "good quality" with Jadad score ≥3 and four studies as "low quality" with Jadad score <3 (Table 1). Consensus was reached by both authors in establishing this level of evidence after independent evaluation of the included studies. A meta-analysis was not presented due to studies heterogeneity and methodological flaws.

### Hydroxychloroquine effect on proteinuria

All the included studies assessed the effect of HCQ on proteinuria. Overall, there was a tendency of HCQ treatment to reduce proteinuria.

Due to different study design and increased heterogeneity, we also performed a subgroup analysis. Thus, in the studies where the control arm was supportive therapy, HCQ significantly reduced proteinuria at 6 months. However, in the studies that compared HCQ to immunosuppressive therapy, we found no difference in proteinuria reduction.

### Hydroxychloroquine effect on GFR

During follow-up, eGFR remained mostly stable in all the five included studies. Moreover, the authors reported similar eGFR between the comparison arms.

### Adverse events

Serious side effects were not reported in any of the included studies.

The most frequent adverse events reported were mucocutaneous, followed by gastrointestinal and anaphylactic reactions (Table 2). Cardiovascular events were seldom seen, palpitations being the only reported complaint.

**Table 2.** Adverse events of HCQ in the studies included in the systematic review.

Adverse events	Gao 2017	Liu 2019	Yang 2019	Tang 2020	Total
	*n* = 14	*n* = 30	*n* = 92	*n* = 26	*N* = 162
Cardiovascular					
Palpitations	0	1	1	0	2
Gastrointestinal					
Liver dysfunction Nausea Diarrhea	0	0	1	0	1
	0	1	1	1	3
	0	0	0	1	1
Ophtalmologic					
Intraocular pressure elevation	0	0	1	0	1
Renal					
eGFR decline	0	2	2	0	4
Neuropsyhiatric					
Dizziness	0	1	0	0	1
Mucocutaneous					
Pruritus Skin pigmentation Desquamation Alopecia	0	1	2	0	3
	0	1	1	1	3
	0	0	1	0	1
	0	0	1	0	1
Anaphylactic					
Dyspnea Rashes	0	0	1	0	1
	1	1	2	0	4
Total n (%)	1 (7)	8 (27)	14 (15)	3 (12)	26 (16)

## Discussions

To the best of our knowledge, we report the first systematic review which describes the hydroxychloroquine effect on proteinuria and eGFR in patients with IgA nephropathy. We found that HCQ treatment in addition to optimized supportive therapy seems to significantly and safely reduced proteinuria. Moreover, our analysis suggests that HCQ could be an alternative therapy for patients with insufficient response to immunosuppression.

IgAN has a relatively benign disease course with up to 30% of patients reaching end-stage kidney disease after 20 to 25 years from diagnosis [16]. In the most recent IgAN studies, the included patients experienced GFR declines between 1.5 and 7 mL/min per year [5,7,8]. Therefore, the management has shifted from immunosuppressive measures to supportive care, since immunosuppression has been shown to have limited efficiency and raised safety concerns in the most recent IgAN trials [5,7].

Due to the IgAN slowly progressive nature, proteinuria has been approved as a reasonably useful surrogate endpoint for effectiveness of treatment progression to ESKD [17].

Established supportive measures in IgAN RAASIs, due to their antiproteinuric effect [18]. Recently, HCQ has emerged as a potential antiproteinuric agent in IgAN.

The most compelling evidence for HCQ comes from Liu et al, who randomly assigned 60 patients at high risk for progressive IgAN to receive HCQ or matching placebo for 6 months [11]. The primary outcome, relative change in proteinuria from baseline to end of study, differed significantly between the two groups, with approximately a 50% decrease in the HCQ group as compared to a 10% increase in the placebo group [11]. However, there were no differences regarding eGFR and hematuria [11].

In our systematic review, HCQ significantly reduced proteinuria in the studies where the control arm was on supportive therapy. While most of the included studies had a short follow-up of six months, Tang et al. studied 180 patients with IgAN who had received HCQ therapy for at least 1 year and reported a 50% decrease in proteinuria in 73% of patients after 12 months [19].

These results suggest that HCQ can be a candidate for treatment of IgAN patients with low-grade proteinuria or those who fail to achieve the target level of proteinuria after RAASI therapy.

Interestingly, we found no differences between HCQ and conventional immunosuppression therapy in proteinuria reduction in the included studies. Therefore, the antiproteinuric effect of HCQ might be similar to that of corticosteroids. Moreover, treatment with HCQ could be safer than immunosuppression in IgAN patients.

In all the included studies, during the follow-up the eGFR remained mostly stable in patients who received HCQ. Also, the authors reported similar eGFR between the comparison arms (Table 1). These results further expand on the reno-protective role of HCQ in IgAN and raise the possibility of slowing the progression of chronic kidney disease (CKD).

### Postulated mechanisms for hydroxychloroquine action in IgA nephropathy

Hydroxychloroquine is considered to have an immunomodulatory rather than an immunosuppressant effect. However, the immune pathways targeted by HCQ are not fully inhibited during treatment. Moreover, the model of HCQ action has been based on *in vitro* studies, thus the relationship between these postulated mechanisms and the clinical response have not been fully elucidated.

**Figure 2.**
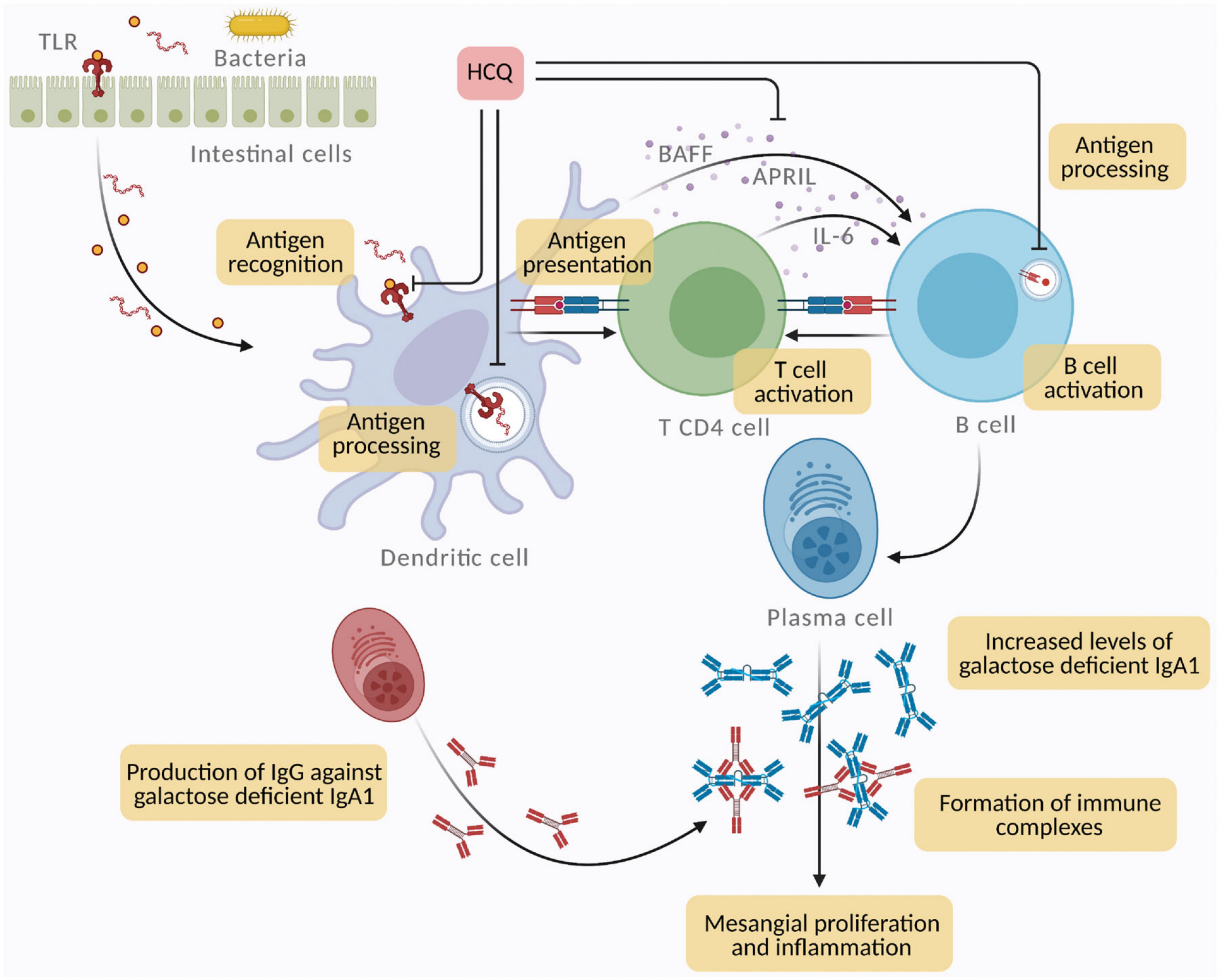
Potential mechanisms for hydroxychloroquine (HCQ) action in IgA nephropathy (IgAN). HCQ can interfere with immune activation at various cellular levels by inhibiting the innate and adaptive immune systems. In IgAN, mucosal Toll like receptor-9 (TLR-9) activation induces B-cell activating factor (BAFF) overexpression in dendritic cells, stimulates the generation of proliferation-inducing ligand (APRIL) and interleukin-6 (IL-6), which act concurrently to stimulate the production of galactose deficient IgA1. HCQ interferes with TLR9 ligand binding and TLR signaling (through lysosomal inhibition), which inhibits TLR-mediated cell activation and cytokine production. Moreover, in antigen presenting cells, such as dendritic cells and B cells, HCQ inhibits antigen processing and subsequent MHC class II presentation to T cells, preventing T cell activation, differentiation and expression of co-stimulatory molecules.

Hydroxychloroquine has direct molecular effects on lysosomal activity, signaling pathways, cytokine production, and immune activation (Figure 2).

Hydroxychloroquine has lysosomotropism. Due to its weak base nature, it accumulates in lysosomes (an acidic compartment) and destabilizes the lysosomal membrane with the release of lytic enzymes inside the cells [20]. This interference with lysosomal activity can inhibit lymphocyte function and lead to anti-inflammatory effects. Moreover, the antigen presentation *via* the lysosomal pathway is also disrupted [21].

Signaling disorders associated with the toll-like receptor (TLR) have been linked to IgAN.

Abnormal innate immune response to mucosal infections could be involved in galactose deficient IgA1 production. In line with this theory, TLR can have a central role since they induce polyclonal proliferation of lymphocytes and circulating immune complex formation [22]. In IgAN, mucosal TLR-9 activation induces B-cell activating factor (BAFF) overexpression in dendritic cells, B-cell expansion, and increased IgA production [23].

Moreover, TLR-9 activation stimulates the generation of a proliferation-inducing ligand (APRIL) and interleukin-6 (IL-6), and, consequently, APRIL and IL-6 act concurrently to stimulate the production of galactose deficient IgA1 [22,23].

Hydroxychloroquine can interfere with TLR signaling [24]. Changes in endosomal pH due to HCQ prevent TLR-9 activation by extracellular stimuli [25,26]. Moreover, HCQ can produce steric blockade by directly binding to nucleic acids in the endosomes, hence inhibiting the interactions between TLR-9 and ligand [24,26].

*In vitro* studies reported that HCQ can indirectly reduce the production of inflammatory cytokines produced by various cell types involved in IgAN [27,28]. Thus, HCQ therapy inhibits the production of tumor necrosis factor-alpha (TNF-alpha), IL-1, and IL-6 by mononuclear cells [24,27].

### Adverse events: drug toxicity

The most frequent adverse events reported in the studies included in our systematic review were mucocutaneous (Table 2). The dermatologic adverse events may be attributed to the strong binding of HCQ to melanin in the skin [29]. Previous reported mucocutaneous complications included nonlight-sensitive psoriasis, alopecia, pruritus, skin and mucosal pigmentation, photosensitivity, and skin eruptions [30]. However, most of these adverse events are often caused by allergic reactions. Moreover, rare cases of critically epidermal necrolysis have been reported [31].

Gastrointestinal complications of HCQ therapy include loss of appetite, diarrhea, nausea, vomiting, and cramps. Interestingly, gastrointestinal adverse events could be due to microbiota alterations induced by HCQ [32]. To prevent gastric discomfort, the HCQ tablet should be taken with a glass of milk or a meal to decrease nausea. Importantly, antacids should not be administrated, as they reduce gastrointestinal absorption.

Cardiotoxicity due to HCQ is rare; the most commonly reported cardiovascular side effects are restrictive or dilated cardiomyopathy and conduction system abnormalities, like atrioventricular block and bundle branch block [33].

The most severe and fearful complication attributed to HCQ therapy is the development of retinopathy. However, in our meta-analysis, there were no reported cases of HCQ induced retinopathy (Table 2).

Five risk factors have been identified as the most important determinants of HCQ related retinopathy: (i) HCQ dose higher than 5 mg/kg actual body weight per day; (ii) prolonged use of HCQ, *i.e.*, more than 10-25 years; (iii) above 600-1000 g cumulative dose of HCQ; (iv) chronic kidney disease stage 3-5; (v) comedication with tamoxifen for more than 6 months [34].

With newer and more sensitive screening techniques, the HCQ retinal toxicity is now more frequently diagnosed than in the past, reaching between 10 and 20% after 20 years of treatment [35].

Of note, most of the treatment duration with HCQ in the studies included in the current systematic reeview was not higher than six months. Since most of the major adverse events of HCQ are reported after more than one year of therapy, longer duration studies are needed for a full characterization of HCQ safety profile in IgAN.

### Hydroxychloroquine dosing consideration in chronic kidney disease

Hydroxychloroquine has a gradual onset of action which might take weeks to reach maximal activity and has prolonged efficacy after discontinuation. These are explained by HCQ pharmacokinetics: long half-life (40 to 60 days), accumulation of dealkylated metabolites, and extensive volume of distribution [24].

Since HCQ it is mainly excreted *via* the kidneys, a 50% dose reduction it is recommended for patients with eGFR <30mL/min [36]. However, dose reduction it is not supported by high-grade evidence. Nevertheless, chronic kidney disease stage 3–5 is considered as one of the most important risk factors for HCQ retinal toxicity [34].

Most of the studies included in the review adjusted the HCQ dose according to eGFR. Besides HCQ dose reduction, other safe recommendations in CKD patients would be to perform more than an annual eye examinations and to avoid drug interactions with anti-arrhythmics (amiodarone, digoxin), antiepileptics, and antipsychotics [24].

### Limitations

Our systematic review has several limitations. Since the authors of the included studies did not respond to our queries regarding missing data and areas of concern, the review was based on published data only. Furthermore, the systematic review was also limited by the methodological quality of studies included and the high heterogeneity in design (only one randomized trial, three case-control studies, one retrospective cohort study, and lack of multicenter studies). Also, all included studies were limited to Chinese patients and most of them had a short follow-up time of only 6 months. Therefore, longer-term trials with more ethnic diversity are required.

## Conclusions

Hydroxychloroquine seems to be an efficient alternative therapy for patients with IgA nephropathy who insufficiently respond to conventional therapy.

However, ethnically diverse randomized controlled studies with long-term follow-up focused on the benefit of proteinuria remission and on the retarding chronic kidney disease progression are needed.

## Data Availability

Data extracted from the included studies in this review are available on request from the corresponding author.

## Declarations

The results presented in this paper have not been published previously in whole or part, except in abstract format.
